# A tuberculosis case mimicking lymphoma

**DOI:** 10.11604/pamj.2014.17.157.3192

**Published:** 2014-03-04

**Authors:** Hilmi Atay, Engin Kelkitli, Mehmet Turgut

**Affiliations:** 1 Van Training and Research Hospital Van, Turkey; 2 Erzurum Region Training and Research Hospital Erzurum, Turkey; 3 319 May's University Medical School Department of Hematology Samsun, Turkey

**Keywords:** Tuberculosis, abdominal mass, lymphoma

## Abstract

Tuberculosis remains a worldwide health problem causing morbidity and mortality. Abdominal tuberculosis is a rare form of the disease. Abdominal form of tuberculosis can mimic other non-infectious diseases. In this report, we presented an abdominal tuberculosis presenting with an intra-abdominal mass lesion and multiple lymphadenopathies that mimics lymphoma.

## Introduction

Tuberculosis (TB) remains a worldwide health problem causing morbidity and mortality particularly in developing and low-income countries. Abdominal TB is a rare form of the disease and also diagnosis may be challenging due to non-specific signs and symptoms of the disease. Abdominal form of TB can mimic other non-infectious diseases such as; inflammatory bowel disease, lymphoma and peritoneal carcinomatosis. Herein, we present a case report with abdominal TB presenting with an intra-abdominal mass lesion and multiple lymphadenopathies that mimics lymphoma.

## Patient and observation

**Figure 1:**
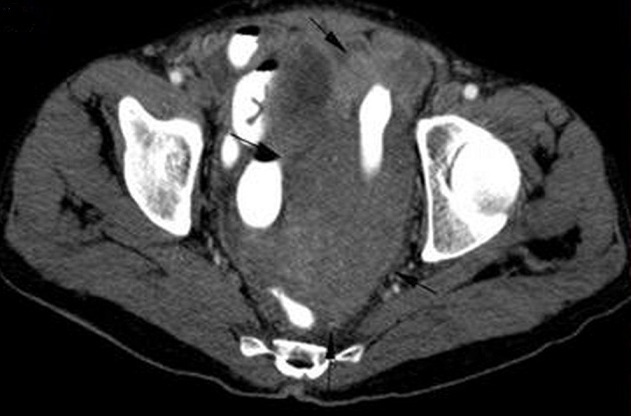
Abdominal computed tomography showing a uniform-density mass lesion of 70 mm that filled retrovesical and left pelvic area also multiple lymph nodes in the retro-vesical, celiac, para-aortic and para-iliac areas

**Figure 2:**
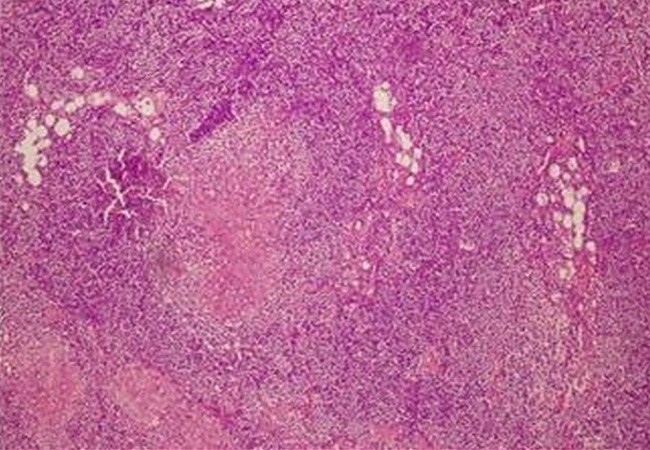
Histological examination of biopsy specimen shows epithelioid cell granulomas, Langhans’ type multinucleated giant cells, and caseous necrosis. Magnification ×100 (Hematoxylin and eosin stain staining)

A 57-year-old male presented with abdominal pain, fatigue, high fever, and weight loss of 6 kg for two months. His previous medical history was unremarkable. Physical examination revealed pain on the left side of the abdomen and hepatosplenomegaly. Laboratory findings were as follows: erythrocyte sedimentation rate 86 mm/h, leukocyte 12600/µl, neutrophile 7200/µl, thrombocyte 510000/µl, monocyte 2100/µL, hemoglobin 10.2 g/dl, hematocrit 30%, MCV 74.6 fl, and lymphocyte 2200/µl. Computed tomography (CT) of the chest showed multiple mediastinal lymphadenopathies (maximum size 15 mm) and bilateral apical fibrotic bands. A uniform-density mass lesion of 70 mm that filled retrovesical and left pelvic area was detected in abdominal CT. The lesion surrounded superior part of sigmoid colon and there were multiple lymph nodes in the retro-vesical, celiac, para-aortic and para-iliac areas (Figure 1). Histological examination of samples that were obtained during diagnostic laparotomy showed epithelioid granulomas, Langhans? type multinucleated giant cells, and caseous necrosis (Figure 2). Lowenstein-Jensen culture of samples confirmed the presence of TB. Antituberculous therapy comprising rifampicin, isoniazid, ethambutol and pyrazinamide was initiated and continued for two months, and the patient was maintained on rifampicin and isoniazid for 6 months. In the sixth month follow-up of patient, cerebrovascular hemorrhage developed. The patient died due to this.

## Discussion

Abdominal TB rarely presents with abdominal mass, so distinguishing from a malignancy is not easy. In this case, abdominal CT findings suggested a malignancy, especially lymphoma. A few TB cases were reported to present with findings mimicking cervical carcinoma [1], over cancer [2], colon cancer [3] and pancreatic cancer [4]. Uncertain origin and invasive characteristics of the large mass lesion were distinctive features in our case report. This is the first case who had abdominal large mass associated with TB mimicking abdominal lymphoma according to published reports.

## Conclusion

Abdominal TB is a rare form of tuberculosis. It can present with symptoms and signs mimicking lymphoma. It should be taken into consideration that TB can present like a malignant abdominal mass in countries with a higher TB prevalence, especially in the developing countries, by physicians and radiologists.
